# Supplementary material for: Polyketide synthase gene clusters in the ericoid mycorrhizal fungus Oidiodendron maius: genome-wide prediction, expression profiling, and functional characterization of knockout mutants
Source: Front Plant Sci. 2026 Sep 16;17:1924430. doi: 10.3389/fpls.2026.1924430 (PMC13623903; doi:10.3389/fpls.2026.1924430)
Supplement: Supplementary file 1 [file SupplementaryFile1.zip › Supplementary Materials/Supplementary Folder 1.zip!/Oidma1_AssemblyScaffolds_Repeatmasked/knownclusterblast/cluster36/jgi.p_Oidma1_44441_mibig_hits.html]

| MIBiG Protein | Description | MIBiG Cluster | MiBiG Product | % ID | % Coverage | BLAST Score | E-value |
| --- | --- | --- | --- | --- | --- | --- | --- |
| AAS90069.1 | AflT | BGC0000010 | Aflatoxin\_biosynthetic\_gene\_cluster | 47 | 87.7551020408 | 467 | 3.2e-131 |
| CAP96439.1 |  | BGC0000420 | Roquefortine\_C\_/\_meleagrin\_biosynthetic\_gene\_cluster | 44 | 89.9659863946 | 462 | 6.1e-130 |
| AAS89998.1 | AflT | BGC0000007 | Aflatoxin\_biosynthetic\_gene\_cluster | 48 | 83.5034013605 | 455 | 1.3e-127 |
| AAS90021.1 | AflT | BGC0000008 | Aflatoxin\_biosynthetic\_gene\_cluster | 47 | 87.7551020408 | 453 | 3.7e-127 |
| AAS90092.1 | AflT | BGC0000006 | Aflatoxin\_biosynthetic\_gene\_cluster | 47 | 87.7551020408 | 451 | 1.4e-126 |
| AAS90046.1 | AflT | BGC0000009 | Aflatoxin\_biosynthetic\_gene\_cluster | 47 | 84.0136054422 | 449 | 5.4e-126 |
| AMR44276.1 | MFS\_multidrug\_transporter | BGC0001398\_c3 | Phomopsins\_biosynthetic\_gene\_cluster | 44 | 89.6258503401 | 448 | 9.1e-126 |
| ADY16699.1 | TqaJ | BGC0001142 | Tryptoquialanine\_biosynthetic\_gene\_cluster | 44 | 86.7346938776 | 442 | 6.5e-124 |
| BAD29973.1 | transporter\_protein | BGC0000676 | Aphidicolin\_biosynthetic\_gene\_cluster | 42 | 91.156462585 | 435 | 1.4e-121 |
| BAE71313.1 | putative\_ABC\_transporter | BGC0000004 | Aflatoxin\_biosynthetic\_gene\_cluster | 47 | 82.4829931973 | 431 | 1.2e-120 |
| ACD39756.1 | major\_facilitator\_superfamily\_transporter | BGC0000076 | Hypothemycin\_biosynthetic\_gene\_cluster | 45 | 85.7142857143 | 430 | 4.4e-120 |
| ACD39765.1 | major\_facilitator\_superfamily\_transporter | BGC0000077 | Hypothemycin\_biosynthetic\_gene\_cluster | 45 | 85.7142857143 | 430 | 4.4e-120 |
| CCE28984.1 | probable\_DHA14-like\_major\_facilitator;\_ABC\_transporter | BGC0001365 | Clapurines\_biosynthetic\_gene\_cluster | 45 | 87.925170068 | 424 | 1.8e-118 |
| CBF87265.1 | MFS\_toxin\_efflux\_pump\_(AflT),\_putative\_(AFU\_orthologue;\_AFUA\_1G12620) | BGC0000680\_c2 | Austinol/dehydroaustinol,\_locus\_1\_biosynthetic\_gene\_cluster | 42 | 83.3333333333 | 393 | 3.5e-109 |
| XP\_002384861.1 | MFS\_multidrug\_transporter,\_putative | BGC0001234\_c2 | 4,4'-piperazine-2,5-diyldimethyl-bis-phenol\_biosynthetic\_gene | 41 | 81.8027210884 | 378 | 2e-104 |
| ACZ66257.1 | APS11 | BGC0000304 | Apicidin\_biosynthetic\_gene\_cluster | 35 | 93.1972789116 | 333 | 5.6e-91 |
| ADI24949.1 | GsfJ | BGC0000070 | Griseofulvin\_biosynthetic\_gene\_cluster | 37 | 86.9047619048 | 325 | 1.5e-88 |
| CBF76046.1 | conserved\_hypothetical\_protein | BGC0001399 | Fellutamide\_B\_biosynthetic\_gene\_cluster | 36 | 83.3333333333 | 321 | 1.7e-87 |
| AAB36607.1 | toxin\_pump | BGC0000370\_c2 | HC-toxin\_biosynthetic\_gene\_cluster | 34 | 91.8367346939 | 313 | 4.6e-85 |
| ADM34144.1 | efflux\_pump | BGC0001084 | Notoamide\_/\_stephacidin\_biosynthetic\_gene\_cluster | 35 | 92.0068027211 | 312 | 7.8e-85 |
| AAD34558.1 | unknown | BGC0000089 | Lovastatin\_biosynthetic\_gene\_cluster | 36 | 87.7551020408 | 308 | 1.5e-83 |
| AGC95323.1 | CurE | BGC0000045 | Dehydrocurvularin\_biosynthetic\_gene\_cluster | 35 | 89.9659863946 | 305 | 1.6e-82 |
| BAC20568.1 | efflux\_pump | BGC0000039 | Compactin\_biosynthetic\_gene\_cluster | 37 | 87.4149659864 | 303 | 4.7e-82 |
| AHV78251.1 | ResE | BGC0001246 | Trans-resorcylide\_biosynthetic\_gene\_cluster | 36 | 82.1428571429 | 301 | 1.8e-81 |
| ABA02247.1 | efflux\_pump | BGC0000098 | Monacolin\_K\_biosynthetic\_gene\_cluster | 36 | 83.3333333333 | 290 | 5.4e-78 |
| EAU36749.1 | predicted\_protein | BGC0000292 | Acetylaranotin\_biosynthetic\_gene\_cluster | 36 | 81.6326530612 | 281 | 1.9e-75 |
| EAQ86390.1 | hypothetical\_protein | BGC0001405 | Chaetoviridin\_/\_chaetomugilin\_biosynthetic\_gene\_cluster | 33 | 73.4693877551 | 236 | 7.1e-62 |
| ctg1\_orf0002 |  | BGC0000688 | Copalyl\_diphosphate\_biosynthetic\_gene\_cluster | 37 | 65.9863945578 | 232 | 1e-60 |
| ACZ57546.1 | predicted\_MFS\_transporter | BGC0000046 | Depudecin\_biosynthetic\_gene\_cluster | 29 | 91.6666666667 | 227 | 3.3e-59 |
| AMY15055.1 | MFS\_transporter | BGC0001339 | Squalestatin\_S1\_biosynthetic\_gene\_cluster | 30 | 84.693877551 | 220 | 5.3e-57 |
| AAD13557.1 | LanJ | BGC0000239 | Landomycin\_biosynthetic\_gene\_cluster | 31 | 80.612244898 | 214 | 3.8e-55 |
| ACS68556.1 | major\_facilitator\_superfamily\_protein | BGC0001026 | NG-391\_biosynthetic\_gene\_cluster | 29 | 88.6054421769 | 214 | 3.8e-55 |
| CAH10123.1 | putative\_transporter | BGC0000268 | Sch47554\_/\_Sch47555\_biosynthetic\_gene\_cluster | 29 | 86.7346938776 | 213 | 6.5e-55 |
| AAF00219.1 | transporter | BGC0000276 | Urdamycin\_biosynthetic\_gene\_cluster | 30 | 80.9523809524 | 213 | 6.5e-55 |
| AAQ08935.1 | putative\_membrane\_transporter | BGC0000224 | Fredericamycin\_biosynthetic\_gene\_cluster | 32 | 72.2789115646 | 202 | 1.1e-51 |
| ACP19369.1 | SaqJ1 | BGC0000267 | Saquayamycin\_Z\_/\_galtamycin\_B\_biosynthetic\_gene\_cluster | 29 | 88.4353741497 | 196 | 6.3e-50 |
| EAA65599.1 | hypothetical\_protein | BGC0000022 | Asperfuranone\_biosynthetic\_gene\_cluster | 30 | 94.5578231293 | 194 | 4.1e-49 |
| AGA37273.1 | efflux\_pump | BGC0000819 | Paraherquamide\_biosynthetic\_gene\_cluster | 36 | 29.5918367347 | 128 | 3.6e-29 |
| ADE34501.1 | SsfR | BGC0000269 | SF2575\_biosynthetic\_gene\_cluster | 25 | 84.0136054422 | 117 | 4.8e-26 |
| ACN69979.1 | efflux\_transporter | BGC0000079 | Indanomycin\_biosynthetic\_gene\_cluster | 26 | 70.9183673469 | 101 | 4.7e-21 |
